# Study on the Anticancer Effect of an Astragaloside- and Chlorogenic Acid-Containing Herbal Medicine (RLT-03) in Breast Cancer

**DOI:** 10.1155/2020/1515081

**Published:** 2020-06-10

**Authors:** Yanchu Li, Xianyong Li, Chen Cuiping, Rong Pu, Yin Weihua

**Affiliations:** ^1^Oncology Department, West China Hospital of Sichuan University, Chengdu, China; ^2^Oncology Department, Chengdu Fuxing Hospital, Chengdu, China; ^3^Research Institution, Chengdu Fuxing Hospital, Chengdu, China

## Abstract

**Background:**

Although surgery, chemotherapy, radiotherapy, and endocrine therapy are widely used in clinical practice for breast cancer treatment, herbal medicines (HMs) are considered as an alternative to palliative treatments because of their coordinated intervention effects and relatively low side effects. Astragaloside (AS) and chlorogenic acid (CGA) are major active ingredients of *Radix Astragali* and *Lonicera japonica*, which have shown antitumorigenic properties in certain cancers, but the role of HMs containing both AS and CGA remains unclear in breast cancer. In this study, we explored an AS- and CGA-containing HM (RLT-03) extracted from *Radix Astragali*, *Lonicerae Japonicae Flos*, *Trichosanthin,* and *Rhizoma imperatae*.

**Methods:**

RLT-03 was extracted using water and n-butanol, and the AS and CGA ingredients in RLT-03 were identified by high-performance liquid chromatography (HPLC) and evaporative light-scattering detector (ELSD). 4T1, EMT6, BT-549, and MDA-MB-231 breast cancer cell lines were used, and an EMT6 xenograft model was established. Cell proliferation, migration, and apoptosis were measured *in vitro*, and tumor volume and weight were observed *in vivo*. The expression of VEGF, EGF, IL-10, TGF-*β*, and CD34 and cell apoptosis in tumors were examined.

**Results:**

RLT-03 inhibited cell viability and induced apoptosis in a dose- and time-dependent manner. *In vivo*, tumor volume and weight were reduced, and the expression of VEGF, EGF, IL-10, TGF-*β*, and CD34 was suppressed in the tumor microenvironment, while cell apoptosis was induced.

**Conclusion:**

RLT-03 exhibited therapeutic effects against breast cancer by regulating the expression of ligands of receptor tyrosine kinases (RTKs) and inflammatory factors. Thus, RLT-03 represents a potential supplementary HM that can be used in breast cancer therapy.

## 1. Introduction

Breast cancer is one of the most common malignant tumors in women. About 1.3 million women are affected by breast cancer every year worldwide, and more than 330,000 women die from it [1, 2]. Surgery, chemotherapy, radiotherapy, and endocrine therapy are widely used in clinical practice, but such therapies have limitations [3, 4]. Therefore, finding a way to suppress cancer and improve the quality of life of patients is particularly important in the treatment and prevention of breast cancer, especially for late-stage patients and those with a poor performance status. Currently, herbal medicines (HMs) are considered as capable of inducing immunomodulatory effects. They protect cancer patients from complications and improve quality of life and survival time [5, 6]. For example, KIOM-C and *Ganoderma lucidum* have been used in the treatment of malignant HT1080 sarcoma and ovarian cancer [7, 8].

Astragaloside (AS) and chlorogenic acid (CGA) are major active ingredients derived from *Radix Astragali* and *Lonicera japonica*, which have shown antitumorigenic properties in certain cancers. In previous studies, *Astragalus*-based HMs have been shown to enhance the efficacy of platinum-based chemotherapy and improve platinum-derived toxicities for late-stage non-small cell lung carcinoma (NSCLC) [9]. In addition, they have been shown to inhibit the viability and invasive potential of MDA-MB-231 breast cancer cells by suppressing the activation of the mitogen-activated protein kinase (MAPK) pathway and downregulating matrix metalloproteases (MMPs)-2 and -9 [10]. Furthermore, several studies have indicated that CGA may act against cancer [11, 12] by regulating the expression of apoptosis-associated genes and causing cell-cycle arrest [13–16]. However, the role of an HM containing both AS and CGA in breast cancer remains unclear.

Therefore, the present study aimed to explore the possible function and mechanism of the astragaloside- and chlorogenic acid-containing HM (RLT-03) in breast cancer progression in vitro and vivo.

## 2. Material and Methods

### 2.1. Cell Lines and Culture

4T1, EMT6, BT-549, and MDA-MB-231 breast cancer cell lines were used. 4T1, BT-549, and MDA-MB-231 cells were cultured in DMEM (Thermo Scientific HyClone, USA), and EMT6 cells were cultured in RPMI 1640 (Thermo Scientific HyClone, USA) containing 10% fetal bovine serum (FBS) and 1% penicillin and streptomycin, at 37°C in an atmosphere of 5% CO_2_.

### 2.2. RLT-03 Medicine Preparation

RLT-03 is a HM developed by the Pharmaceutical Department of Chengdu Fuxing Hospital and has been used for the treatment of breast cancer. The theoretical guides behind RLT-03 formula were the traditional Chinese medicine theory “Jun, Cheng, Zuo, and Shi (Monarch, Minister, Assistant, and Guide)” and the *Malignant Tumor Projection Theory (MTPT)*. This HM is prepared mainly from the extracts of *Radix Astragali* (Chinese name: Huang Qi), *Lonicerae Japonicae Flos* (Chinese name: Jin Yinhua), *Trichosanthin* (Chinese name: Tian Huafen), and *Rhizoma imperatae* (Chinese name: Bai Maogen) by water and n-butanol. The extract was dried in rotary evaporators and a vacuum drying chamber; the dried fractions were stored at 26°C in a dryer.

### 2.3. Elements Analysis of RLT-03

High-performance liquid chromatography (HPLC) and an evaporative light-scattering detector (ELSD) were used to identify AS and CGA in RLT-03. Ten microliters of a sample of RLT-03 (5.07 mg/ml) and standard samples were injected into the HPLC system (Waters 2695 and Waters 2996 Diode Array Detector, USA) and ELSD (Sedere Sedex75, France), respectively.

### 2.4. MTT Assay

Cell viability was quantitated by the MTT assay (Sigma-Aldrich, St Louis, MO, USA). 4T1, EMT6, BT-549, and MDA-MB-231 cells (5 × 10^3^ cells/well) were seeded in 96-well plates and exposed to different concentrations of RLT-03 (0.625, 1.25, 2.5, 5.0, and 7.5 mg/ml for 4T1 cells; 1.0, 1.5, 2.0, 2.5, 3.0, and 3.5 mg/ml for EMT6, BT-549, and MDA-MB-231 cells). After 24 hours of incubation, 20 *μ*l of 5 mg/ml MTT solution was added to each well and the plate was further incubated at 37°C for 4 hours. Thereafter, the medium was aspirated and 200 *μ*l of DMSO (Sigma) was added to each well [17]. After the formazan crystals had dissolved, the absorbance was determined spectrophotometrically at 492 nm on an INFINITE F50 microplate reader (TECAN, Austria). This procedure was replicated thrice.

### 2.5. Crystal Violet Assay

4T1, EMT6, BT-549, and MDA-MB-231 cells were seeded in 24-well plates at 1.0 × 10^4^ cells per well and incubated for 24 hours. Then, cells were exposed to different concentrations of RLT-03 (1.25, 2.5, 3.0, and 4.0 mg/ml for 4T1 cells; 1.0, 1.5, 2.0, and 3.0 mg/ml for EMT6 and BT-549 cells; 0.5, 1.0, 1.25, and 1.5 mg/ml for MDA-MB-231 cells) for 72 hours. After fixation with 4% paraformaldehyde for 30 min, the cells were stained with crystal violet solution for 2 hours. Images were taken after washing the cells with PBS. This procedure was replicated thrice.

### 2.6. Cell Scratch Assay

Cell migration ability was quantitated by the cell scratch assay. Approximately 2 × 10^4^ cells were aliquoted into each well of a 6-well plate, and a microscope was used the following day to confirm that each well was coated with cells. A 1 mL pipette tip was used to scratch cells from the bottom of the well, and the plates were washed with phosphate-buffered saline three times to remove the displaced scratched cells. Cells were exposed to different concentrations of RLT-03 (2.0 and 3.0 mg/ml for 4T1 and BT-549 cells; 1.25 and 2.0 mg/ml for MDA-MB-231 cells; 1.5 and 2.0 mg/ml for EMT6 cells). Cells were cultivated simultaneously in an incubator at 37°C under 5% CO_2_. Images of the samples were captured at 0, 24, and 48 h by Nikon TS100 (Nikon, Japan). This procedure was repeated thrice.

### 2.7. Xenograft Breast Cancer Model and Treatment

Female SPF-Balb/c mice weighing 21 ± 1.2 g were obtained from the Chengdu Dossy Experimental Animals Co. Ltd. (Chengdu, China). This animal study was approved by the Animal Care and Use Committee. EMT6 cells (1 × 10^6^ cells/mouse) were transplanted into the right dorsal side of each mouse. Sixteen tumor-bearing mice were divided into two groups (*n* = 8 each). Saline was administered to the blank control group by oral gavage, while 20.0 mg/g of RLT-03 was administered to the sample group by oral gavage for 21 days. The tumor volume and weight were recorded at three-day intervals. Tumor volume was calculated using the following formula: volume (mm^3^) = width^2^ × length/2.

### 2.8. Enzyme-Linked Immunosorbent Assay (ELISA)

The ELISA kits (20140603DE and 20140603DV, Abcam) were used to detect the levels of the IL-10 and IL-12 cytokines in the serum. Cytokine levels were analyzed by measuring the absorbance at 450 nm on Multiskan Mk3 (Thermo Fisher, USA).

### 2.9. Hematoxylin-Eosin Staining Assay

Tumor tissues were dissected from the implanted mice, fixed in 10% buffered formalin for 24 hours, and embedded in paraffin. Tissue sections were cut at 5 *μ*m thickness and stained with hematoxylin-eosin. Images were acquired by Motic BA400 (Motic, China).

### 2.10. TUNEL Assay

TUNEL assay (Roche, 10279600 kits) was performed to evaluate apoptosis in tumor sections. Tumor sections were dewaxed at 26°C. Sections were treated with 3% H_2_O_2_ and incubated with equilibration buffer and terminal deoxynucleotidyl transferase enzyme. Finally, sections were incubated with antidigoxigenin-peroxidase conjugate. Tissue peroxidase activity was evaluated through DAB application. Images were acquired by Motic BA400 (Motic, China).

### 2.11. Immunohistochemistry Assay

Tissues were fixed in 10% buffered formalin and embedded in paraffin. Paraffin sections of tumor tissues were prepared, dewaxed by dimethyl-benzene, and hydrated with different concentrations of ethanol (100%, 95%, 85%, 70%, and 50%). The following steps were performed: blocking of endogenous peroxidase activity in 3% H_2_O_2_ solution, unmasking of the antigenic epitope with citrate buffer, incubation with blocking buffer for blocking, and incubation with primary antibody (VEGF (Bioss, BS1665R), CD34 (Bioss, BS5085R), TGF-*β*1(Bioss, BS0086R), IL-10 (Bioss, BS6761R), and EGFR (Bioss, BS1007R)) and secondary antibody (horseradish peroxidase-labeled goat rabbit IgG (H + L) (Beyotime, A0208)). Lastly, the DAB (Beyotime) substrate solution was applied to reveal the color of antibody staining. Images were acquired by an Motic BA400 microscope (Motic, China). The expression of cytokines was analyzed by Image-Pro Plus 6.0 (Media Cybernetics, USA).

### 2.12. Western Blot Assay

Tumor tissues were lysed in lysis buffer and then centrifuged at 15,000 rpm for 15 min at 4°C. Protein concentration was determined using the BCA kit (Beyotime). A total of 50 *µ*g of protein was subjected to 8–10% SDS-PAGE and transferred to a PVDF membrane (Merck Millipore, USA). The membranes were blocked for 1 hour at 26°C with 5% bovine serum albumin containing 0.1% Tween-20 and incubated with the primary antibodies (EGF, VEGF, and TGF-*β*) (Beyotime) (diluted by 1 : 1000) overnight at 4°C. Then, the membranes were washed with TBST three times and incubated with the corresponding secondary antibody (diluted by 1 : 5000) at 37°C for 2 hours. The membranes were then washed again, and the proteins were visualized using an enhanced chemiluminescence assay kit (Beyotime). Images were captured by JY-Clear ECL (JUNYI, China).

### 2.13. Flow Cytometry Assay

In vitro, 4T1, EMT6, and BT-549 cells (5 × 10^5^ cells/well) were seeded in 6-well plates. Cells were exposed to RLT-03 (dose = IC_50_) for 24 hours before being collected. Cells were stained with propidium iodide and an Annexin V kit (Yeasen, China) for 15 min in the dark. Samples were examined using a Guava® EasyCyte plus flow cytometer (Merck, USA).

### 2.14. Statistical Analysis

Results are expressed as means. The difference between mean values was assessed by the *t*-test using Prism GraphPad 8.0. A *p* value of <0.05 was considered to indicate a statistically significant difference.

## 3. Results

### 3.1. RLT-03 Contains Astragaloside and Chlorogenic Acid

**Figure 1:**
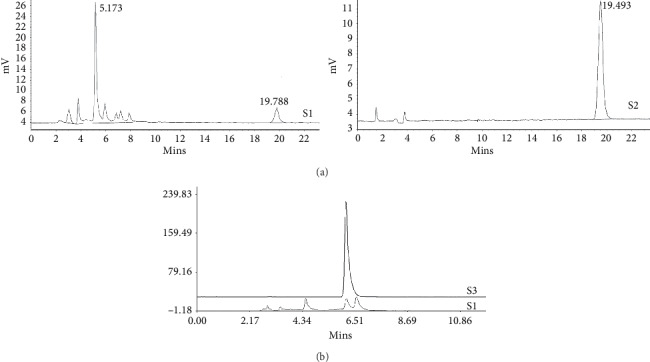
RLT-03 contains astragaloside (AS) and chlorogenic acid (CGA). (a) ELSD analysis of RLT-03 HM. AS was identified in RLT-03. S1 represents AS at the retention time of 19.788 min, which is consistent with the astragaloside standard sample. (b) HPLC analysis of RLT-03 medicine. S1 represents CGA at the retention time of 6.197/6.613 min which is consistent with the chlorogenic acid standard sample (S1, the RLT-03 sample; S2, the AS standard sample; S3, the CGA standard sample).

The elements of RLT-03 were analyzed by HPLC and ELSD. The retention time of the peaks in the HPLC and ELSD corresponded to those of astragaloside and chlorogenic acid, respectively (Figure 1).

### 3.2. RLT-03 Inhibited Breast Cancer Cell Proliferation

**Figure 2:**
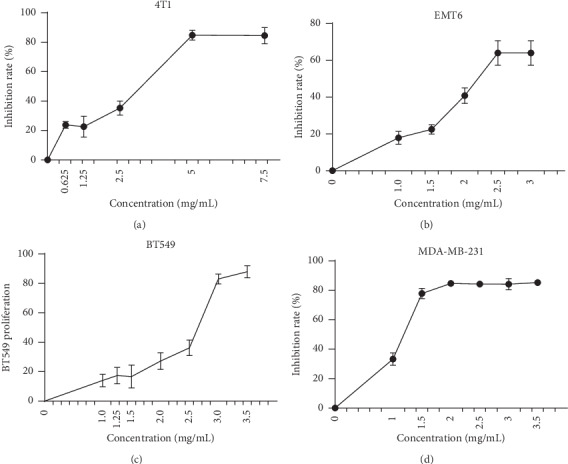
Analysis of cell viability by the MTT assay. RLT-03 inhibited 4T1, EMT6, BT-549, and MDA-MB-231 cell proliferation in a concentration-dependent manner (*p* < 0.05^*∗*^). The IC_50_ of RLT-03 in 4T1, EMT6, BT-549, and MDA-MB-231 cells was 2.387 mg/ml, 2.002 mg/ml, 2.583 mg/ml, and 0.638 mg/ml, respectively.

**Figure 3:**
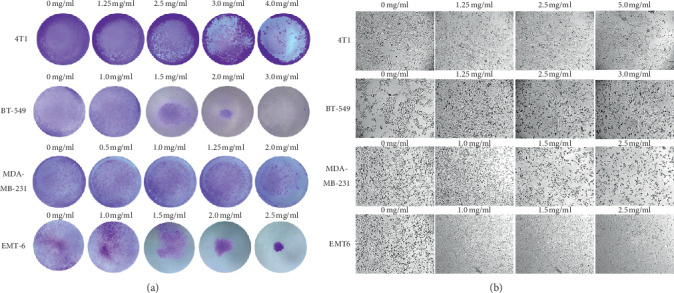
(a) Analysis of cell viability by the crystal violet assay; 4T1, BT-549, MDA-MB-231, and EMT6 cells were exposed to RLT-03 for 72 h, and cell viability was inhibited in a concentration-dependent manner. (b) 4T1, EMT6, BT-549, and MDA-MB-231 cell morphology changed, and cell death was observed after exposure to RLT-03 for 72 h.

The MTT assay was used for analyzing cell proliferation after exposure to different concentrations of RLT-03. The results demonstrated that cell proliferation was inhibited in a concentration-dependent manner. As the RLT-03 concentration increased, breast cancer cell proliferation decreased (Figure 2). The inhibition rates were up to 84.56 ± 5.48%, 64.01 ± 6.67%, 87.92 ± 4.14%, and 85.28 ± 0.86% for 4T1, EMT6, BT-549, and MDA-MB-231 cells, respectively. The IC_50_ of RLT-03 in 4T1, EMT6, BT-549, and MDA-MB-231 cells was 2.387 mg/ml, 2.002 mg/ml, 2.583 mg/ml, and 0.638 mg/ml, respectively. Furthermore, the crystal violet assay showed that cell viability was notably suppressed (Figure 3(a)), and the morphology of 4T1, BT-549, MDA-MB-231, and EMT6 cells changed (Figure 3(b)).

### 3.3. RLT-03 Inhibited Breast Cancer Cell Migration

**Figure 4:**
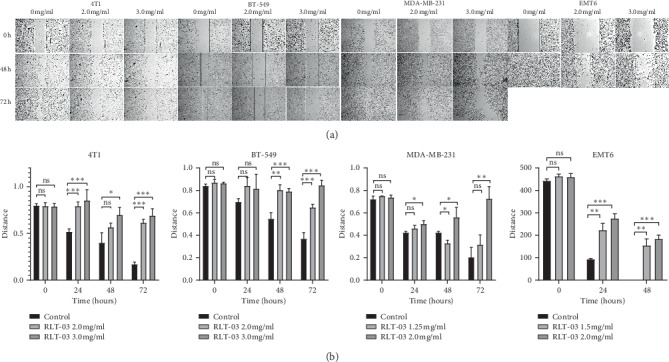
Analysis of cell migration by the scratch assay. Scratch distance is shown in the different groups of cells. Cells treated with and without RLT-03 were observed at 0, 24, 48, and 72 h. RLT-03 inhibited cell migration in a concentration- and time-dependent manner. Magnification: 40x (*p* < 0.01^*∗∗*^).

A difference in the rate of scratch closure areas of 4T1, MDA-MB-231, BT-549, and EMT6 cells following RLT-03 treatment for 72 h was observed. In 4T1 cells, the migration inhibition rates were 71.28% at 2.0 mg/ml (*p* < 0.001^*∗∗∗*^) and 84.41% at 3.0 mg/ml (*p* < 0.001^*∗∗∗*^); in BT-549 cells, the migration inhibition rates were 54.75% at 2.0 mg/ml (*p* < 0.001^*∗∗∗*^) and 96.26% at 3.0 mg/ml (*p* < 0.001^*∗∗∗*^); in MDA-MB-231 cells, the migration inhibition rates were 18.29% at 1.25 mg/ml (*p* > 0.05) and 97.65% at 2.0 mg/ml (*p* < 0.01^*∗∗*^); in EMT6 cells, the migration inhibition rates were 33.45% at 1.5 mg/ml (*p* < 0.01^*∗∗*^) and 39.99% at 2.0 mg/ml (*p* < 0.001^*∗∗∗*^). The results showed that in the cells treated with RLT-03, the scratch distance was significantly wider than in the control group. In BT-549 and MDA-MB-231 cells treated with a high concentration of RLT-03, cell death was observed rather than cell migration. The data indicated that RLT-03 inhibited cell migration in a concentration- and time-dependent manner (Figure 4).

### 3.4. RLT-03 Induced Breast Cancer Cell Apoptosis

**Figure 5:**
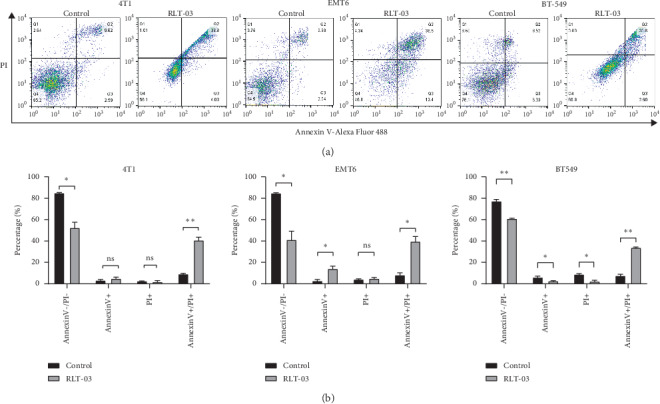
Flow cytometry assay results of RLT-03 induced breast cancer cell apoptosis. RLT-03 induced an increase in the proportion of Annexin V-positive and Annexin V/PI-positive 4T1, EMT6, and BT-549 cells, and the IC_50_ dose of RLT-03 mainly induced late apoptosis (*p* < 0.05^*∗*^).

The ability of RLT-03 to induce apoptosis of breast cancer cells was examined. The flow cytometry assay (FCM) showed that the percentages of Annexin V-positive and Annexin V/PI-positive cells were significantly increased after treatment with RLT-03. In 4T1, EMT6, and BT-549 cells, the Annexin V-positive rates were up to 4.97 ± 1.32% (*p* > 0.05), 14.11 ± 2.42% (*p* > 0.05^*∗*^), and 2.68 ± 0.26% (*p* < 0.05^*∗*^), and the Annexin V/PI positive rates were up to 40.79 ± 2.81% (*p* < 0.01^*∗∗*^), 39.70 ± 4.52% (*p* < 0.05^*∗*^), and 33.97 ± 0.52% (*p* < 0.01^*∗∗*^), respectively. The percentage of Annexin V-positive and Annexin V/PI-positive cells was significantly increased compared to the control group (Figure 5).

### 3.5. Effect of RLT-03 on Tumor Growth in EMT6 Tumor-Bearing Mice

**Figure 6:**
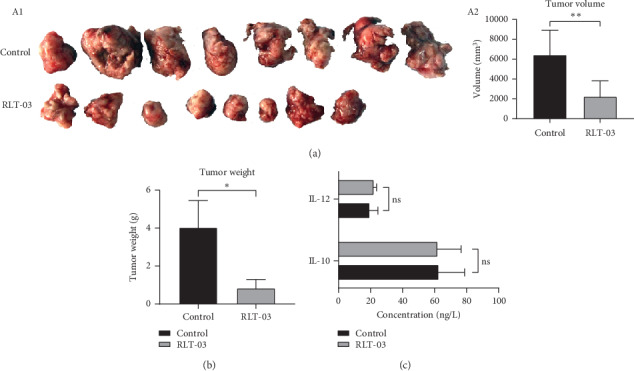
The effect of RLT-03 on tumor volume and weight. The EMT6 breast cancer xenograft model was established. Sixteen tumor bearing mice were divided into two groups (*n* = 8 each). (a) The tumor volume of the RLT-03 group was significantly reduced (*p* < 0.05^*∗*^). (b) The tumor weight of the RLT-03 group was significantly decreased (*p* < 0.05^*∗*^). (c) ELISA of IL-10 and IL-12 expressions in the serum. IL-10 and IL-12 expressions in the serum were the same as the control group (*p* > 0.05).

The tumor weight and volume of the EMT6 tumor-bearing mice in the RLT-03 group (dose = 20 mg/g) were significantly reduced compared with the control group. The inhibition rate of the tumor weight and volume reached to 79.4% (4.03 ± 1.43 g vs. 0.84 ± 0.45 g; *p* < 0.05^*∗*^) and 65.28% (6432.73 ± 2487.95 vs. 2233.19 ± 1588.20 mm^3^; *p* ≤ 0.01^*∗∗*^), respectively. However, IL-10 and IL-12 expression in the serum were not different compared to the control group (*p* > 0.05) (Figure 6).

### 3.6. Influence on Expression of Cytokines and Cell Apoptosis in Tumor Microenvironment

**Figure 7:**
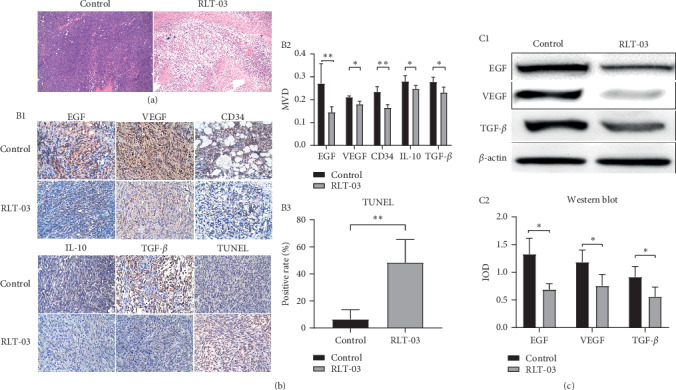
The effect of RLT-03 on tumor tissue necrosis and expression of RTK ligands and inflammatory factors. (a) HE staining. The RLT-03 group exhibited tumor tissue necrosis. (b) CD34, EGF, VEGF, IL-10, and TGF-*β* expression and TUNEL staining were examined in tumor tissue. The expression of CD34, EGF, VEGF, IL-10, and TGF-*β* was inhibited, and RLT-03 induced tumor cell apoptosis (*p* < 0.05^*∗*^). Magnification: 400x. (c) As shown by western blotting, EGF, VEGF, and TGF-*β* protein expressions were inhibited in tumor tissue (*p* < 0.05^*∗*^).

HE staining is shown in Figure 7(a), tumor tissue necrosis was detected in the RLT-03 group compared with the control group. Meanwhile, according to the IHC assay, the integrated optical density (IOD) of EGF, VEGF, CD34, IL-10, and TGF-*β* expression was 0.1451 ± 0.0241 (*p* < 0.01^*∗∗*^), 0.1804 ± 0.0132 (*p* < 0.01^*∗∗*^), 0.1647 ± 0.0142 (*p* < 0.01^*∗∗*^), 0.2474 ± 0.0151 (*p* < 0.05^*∗*^), and 0.2311 ± 0.0244 (*p* < 0.05^*∗*^) in the RLT-03 group compared to 0.2704 ± 0.0872, 0.2109 ± 0.0056, 0.2346 ± 0.0231, 0.2804 ± 0.0247, and 0.2777 ± 0.0209 in the control group, respectively. The positive rate of the TUNEL test in the RLT-03 group was 48.38 ± 17.05% compared to 6.50 ± 7.03% in the control group (*p* < 0.01^*∗∗*^) (Figure 7(b)). Western blot results indicated that the expression of VEGF, EGF, and TGF-*β* was significantly inhibited in vivo (*p* < 0.05^*∗*^). (Figure 7(c)). According to the results, RLT-03 inhibited the expression ligands of receptor tyrosine kinase (RTK) and inflammation factors and induced tumor cell apoptosis.

## 4. Discussion

Breast cancer is one of the most common malignant tumors in women. WHO statistics have shown that about 330,000 patients worldwide die from breast cancer each year [1]. In China, the incidence of breast cancer presents a gradually increasing trend [2]. Therefore, discovering effective drugs for late-stage breast cancer is of utmost importance. Although current therapies for breast cancer, such as chemotherapy, radiotherapy, and targeted therapy, are effective, these therapies have various side effects, are not suitable treatments for late-stage patients, and cannot be used in palliative care [3, 18]. Recently, HMs have been suggested as suitable anticancer drugs for palliative care. Thus, based on the traditional Chinese medicine theory (TCM) and the combined application of multiple components of Compound Chinese Traditional Medicine (CCTM) [19], the combination of nearly 100,000 compounds has been used for clinical prevention and treatment of various diseases [20,21], and the effects of combined HMs for treating breast cancer has been suggested [22, 23]. Furthermore, modernization of Chinese medicine is not just its westernization. In this study, we applied the *Jun, Cheng, Zuo, and Shi* (Monarch, Minister, Assistant, and Guide) theory and *Malignant Tumor Projection Theory (MTPT)* as a theoretical guide behind the formulation of RLT-03 and explored the functions of its chemical components by applying molecular biology experiments in vitro and vivo.

Recently, the inhibitory function of the HM on cancer growth, invasion, and metastasis have been shown to be mediated through angiogenesis, cell differentiation, cell apoptosis, cytotoxic function, and immunity regulation [20, 24, 25]. For example, Kanglaite injection, Kushen injection, and Jinlong capsule have shown clinical efficacy on preventing disease progression with low toxicity [26]. Meanwhile, AS and CGA are major active ingredients from *Radix Astragali* and *Lonicera japonica*, respectively, which have shown antitumorigenic properties in certain cancers [27, 28]. Individually, AS has been reported to have anti-inflammatory, anticancer, antioxidative, and immune-regulatory effects [29, 30]. Consistent with previous studies, AS ameliorated cancer-associated inflammation, decreased the expression of inflammatory factors such as TGF-*β* and IL-10, and suppressed M2 macrophage polarization when administered to treat lung cancer [29]. Moreover, CGA has also been independently reported as having potential for tissue protection and cancer treatment. Studies have indicated that CGA could induce the Nrf2/ARE antioxidant system in hepatic cells [31, 32] and protect the JB6 cell line against environmental carcinogen-induced carcinogenesis though NF-kappa B and MAPK pathways [33].

However, the literature lacks investigations focused on the direct effects of an HM that contains both AS and CGA on breast cancer. As expected, RLT-03 inhibited the growth of breast cancer in vitro and in vivo, which were demonstrated by MTT, scratch, and flow cytometry assays, as well as H&E and TUNEL staining. Besides, western blot and immunohistochemistry assays indicated that RLT-03 significantly inhibited the expression of VEGF, EGF, CD34, IL-10, and TGF-*β* related to the angiogenesis and inflammatory regulation of breast cancer. Moreover, compared to chemotherapy, RLT-03 showed limited pathological toxicity. Recently, RLT-03 has been approved for clinical use by Sichuan Food and Drug Administration, China.

## 5. Conclusion

RLT-03 exhibited prominent therapeutic effects on breast cancer mainly via the regulation of cell proliferation, apoptosis, and the tumor-microenvironment through the downregulation of the expression of RTK ligands and inflammatory factors. Thus, RLT-03 represents a useful and an alternative supplementary medicine for the late-stage and poor performance breast cancer patients, who could not tolerate or refuse second-/third-line therapy. The potential therapeutic effects of RLT-03 may be further ascertained in clinical trials.

## Data Availability

No raw data were used from published articles.
