# Correction to “Study on the Anticancer Effect of an Astragaloside- and Chlorogenic Acid-Containing Herbal Medicine (RLT-03) in Breast Cancer”

**DOI:** 10.1155/ecam/9832548

**Published:** 2025-10-21

Y. Li, X. Li, C. Cuiping, R. Pu, and Y. Weihua, “Study on the Anticancer Effect of an Astragaloside- and Chlorogenic Acid-Containing Herbal Medicine (RLT-03) in Breast Cancer,” *Evidence-Based Complementary and Alternative Medicine* 2020 (2020): 1515081, https://doi.org/10.1155/2020/1515081.

In the article, there are errors in Figure 1(b) relating to an erroneous duplication of several panels during the production process. The correct Figure 1 is shown below:

We apologize for these errors.

## Figures and Tables

**Figure 1:**
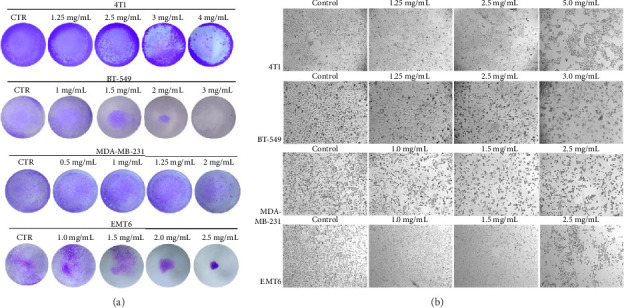
(a) Analysis of cell viability by the crystal violet assay; 4T1, BT-549, MDA-MB-231, and EMT6 cells were exposed to RLT-03 for 72 h, and cell viability was inhibited in a concentration-dependent manner. (b) 4T1, EMT6, BT-549, and MDA-MB-231 cell morphology changed, and cell death was observed after exposure to RLT-03 for 72 h.

